# Association between hypertensive retinopathy and left ventricular hypertrophy

**DOI:** 10.6026/973206300221457

**Published:** 2026-03-31

**Authors:** Mani Shankar Pandey, Jyoti Mani Shankar Pandey

**Affiliations:** 1 Department of Cardiology, Nalanda Medical College and Hospital, Patna, Bihar, India; 2 Department of Ophthalmology, Nalanda Medical college and Hospital, Patna, Bihar, India

**Keywords:** Fundoscopic examination, hypertensive retinopathy (HR), left ventricular hypertrophy (LVH), non-invasive markers, hypertension

## Abstract

The unclear relationship between hypertensive retinopathy (HR) and left ventricular hypertrophy (LVH) in hypertensive patients necessitates further investigation. Therefore, it is of interest to examine the association between hypertensive retinopathy (HR) and left ventricular hypertrophy (LVH) in patients with hypertension. The study included patients diagnosed with hypertension, assessing the presence of HR using fundoscopic examination and LVH through echocardiography. Results revealed a significant correlation between the severity of hypertensive retinopathy and the presence of LVH, suggesting that HR may serve as an early marker for LVH in hypertensive patients. Thus, we show the potential of HR as a non-invasive indicator for cardiovascular risk in hypertension management.

## Background:

Hypertension, a major risk factor for cardiovascular diseases, affects millions of people worldwide. It is often called the "silent killer" due to its asymptomatic nature, which can lead to severe complications if left untreated [1]. Chronic high blood pressure can lead to a variety of systemic consequences, including damage to the heart, kidneys and blood vessels. Two significant manifestations of hypertension-related organ damage are left ventricular hypertrophy (LVH) and hypertensive retinopathy (HR) [2]. Both of these conditions are indicative of end-organ damage and serve as markers for the severity of hypertension. Understanding the relationship between these two conditions could provide valuable insights into the early detection and management of hypertensive cardiovascular complications [3]. Left ventricular hypertrophy refers to the thickening of the left ventricle's walls, often resulting from the heart having to work harder to pump blood against the increased pressure caused by chronic hypertension [4]. LVH is associated with adverse outcomes, including increased risk for heart failure, arrhythmias and even sudden cardiac death. It is diagnosed using imaging techniques such as echocardiography, which can reveal structural changes in the left ventricle. LVH is also closely linked with increased left ventricular mass and diastolic dysfunction, contributing to the progression of heart failure with preserved ejection fraction (HFpEF) [5]. On the other hand, hypertensive retinopathy is a condition that reflects changes in the retina due to chronic high blood pressure. It is classified into different stages, ranging from mild retinal vessel narrowing and arteriovenous nicking to more severe changes such as retinal hemorrhages, exudates and optic disc swelling [6]. HR can be detected through a fundoscopic eye examination, making it one of the few conditions where the retina serves as a direct indicator of systemic vascular health. Hypertensive retinopathy is associated with an increased risk of cardiovascular events, including stroke and heart failure and serves as a marker for poor long-term prognosis in hypertensive patients [7]. Despite the recognized impact of both LVH and HR on cardiovascular health, the relationship between these two conditions has not been well elucidated.

Several studies have shown that the presence of HR is a reflection of the severity of hypertension and may correlate with increased arterial stiffness, a key factor in the development of LVH [8]. However, there is limited research on whether HR can predict the development or severity of LVH, or if the two conditions share common underlying mechanisms related to prolonged hypertension [9]. In clinical practice, LVH and HR are often viewed as independent entities, with treatment strategies typically addressing them separately. However, if these two conditions are found to be closely associated, it could lead to a more integrated approach to managing hypertensive patients, focusing on early detection and prevention of both retinal and cardiac complications [10]. Understanding this potential association could also shed light on the pathophysiology of hypertension-induced organ damage and open new avenues for research on targeted therapeutic strategies [11]. Therefore, it is of interest to describe the clinical implications of platform switching in preserving marginal bone height, evaluate its comparative effectiveness and identify the conditions under which it provides the most significant benefit for long-term implant stability and esthetic outcomes.

## Methodology:

This study was a prospective cohort study at Department of Cardiology, Nalanda Medical College and Hospital, Patna, Bihar, India for one year designed to explore the association between hypertensive retinopathy (HR) and left ventricular hypertrophy (LVH) in patients with hypertension. The study included hypertensive patients aged 18 years and older, who were diagnosed based on standard clinical guidelines (systolic blood pressure ≥ 140 mmHg or diastolic blood pressure ≥ 90 mmHg). Exclusion criteria included patients with a history of diabetes, renal disease, heart failure, previous myocardial infarction, or those with other ocular conditions affecting the retina such as diabetic retinopathy or retinal vascular occlusion. Participants with conditions that could confound the relationship between HR and LVH, such as congenital heart disease or severe systemic illnesses, were also excluded. Patients were recruited from outpatient clinics at a tertiary care hospital, ensuring a diverse cohort of both newly diagnosed and long-term hypertensive individuals. After obtaining informed consent, each participant underwent a comprehensive clinical evaluation, including medical history, blood pressure measurement and laboratory tests. Blood pressure was measured using standard protocols, with three readings taken at 5-minute intervals and the average of these readings was recorded as the participant's baseline blood pressure. A detailed physical examination, including assessment of other cardiovascular risk factors (e.g., smoking, obesity, family history of heart disease), was also performed.

The primary outcome of the study was the association between hypertensive retinopathy (HR) and left ventricular hypertrophy (LVH), while secondary outcomes included the severity of HR, measured via fundoscopic eye examination and the degree of LVH, assessed by echocardiography. Fundoscopic eye exams were conducted by trained ophthalmologists to evaluate the presence and severity of hypertensive retinopathy, classified according to the Keith-Wagener-Barker scale into four stages (mild, moderate, severe and malignant). For the echocardiographic evaluation of LVH, left ventricular mass index (LVMI) was measured using the Devereux formula and LVH was defined as an LVMI greater than 115 g/m^2^in men and 95 g/m^2^ in women. Echocardiography was performed by trained cardiologists using standard transthoracic methods and all measurements were recorded and reviewed by a blinded cardiologist to ensure consistency and accuracy. The study followed participants for 6 months, during which regular follow-up visits every 3 months allowed for the monitoring of blood pressure control and any progression of HR or LVH. Participants were also asked about any new cardiovascular symptoms or events, such as chest pain, dyspnea, or dizziness, during each follow-up visit. Data from these visits, along with the baseline measurements, were compiled for analysis. Sample Size Calculation was conducted to ensure sufficient statistical power for detecting a moderate effect size. Using a two-tailed test with an alpha level of 0.05, a power of 80% and an estimated correlation coefficient of 0.3 between HR and LVH (moderate effect size), the required sample size was calculated to be approximately 120 participants. To account for potential dropouts, non-compliance, or incomplete data, the sample size was inflated by 20%, resulting in a final target sample size of 144 participants. Data analysis was performed using SPSS version 25. Descriptive statistics were used to summarize the demographic and clinical characteristics of the study population. The primary association between HR and LVH was assessed using chi-square tests for categorical variables and Pearson's correlation or Spearman's rank correlation for continuous variables. Multivariable logistic regression was used to assess the independent association between HR and LVH, adjusting for potential confounders such as age, sex, duration of hypertension and blood pressure control. The results were considered statistically significant at a p-value of < 0.05. Ethical approval for this study was obtained from the institutional review board (IRB) of the hospital and all participants provided written informed consent prior to their inclusion in the study.

## Results:

A total of 144 hypertensive patients were enrolled in the study, with a mean age of 58 ± 12 years. Of these, 65% were male and the remaining 35% were female. The study cohort was categorized based on the presence or absence of hypertensive retinopathy (HR) and left ventricular hypertrophy (LVH). Table 1 (see PDF) summarizes the baseline demographic and clinical characteristics of the participants. At baseline, hypertensive retinopathy was present in 45% of the participants, with varying degrees of severity. The most common stage was mild HR (stage 1), which was observed in 30% of the cohort, followed by moderate HR (stage 2) in 10% of patients and severe HR (stage 3) in 5%. Only 2% of participants had malignant HR (stage 4). Table 2 (see PDF) details the distribution of hypertensive retinopathy stages among participants. Regarding left ventricular hypertrophy (LVH), LVH was found in 40% of the study population, with severe LVH being the most prevalent (25%), followed by mild to moderate LVH (15%). The presence of LVH was strongly correlated with increasing age and the duration of hypertension. Table 3 (see PDF) presents the distribution of LVH based on the left ventricular mass index (LVMI) and other clinical parameters. The analysis showed a significant association between the severity of hypertensive retinopathy and the presence of left ventricular hypertrophy. Among participants with severe HR (stage 3), 70% had LVH, compared to 30% in those with mild HR (stage 1). The prevalence of LVH increased progressively with the severity of HR, as shown in Table 4 (see PDF). Chi-square analysis confirmed that the presence of hypertensive retinopathy was significantly associated with the presence of LVH (p < 0.01). Over the course of the 6-month follow-up, patients who had poorly controlled blood pressure (systolic BP> 160 mmHg or diastolic BP > 100 mmHg) were more likely to show progression of both hypertensive retinopathy and left ventricular hypertrophy. Among those with poorly controlled blood pressure, 40% showed an increase in the severity of HR and 30% had worsening of LVH (Table 5 - see PDF). In contrast, patients with well-controlled blood pressure (systolic BP < 140 mmHg) had a significantly lower incidence of HR progression (10%) and LVH worsening (5%). A multivariate logistic regression analysis was conducted to assess the independent factors influencing the presence of LVH. The analysis revealed that hypertensive retinopathy (OR = 2.5, 95% CI 1.8-3.2), age (OR = 1.04, 95% CI 1.02-1.07) and duration of hypertension (OR = 1.15, 95% CI 1.08-1.23) were significant independent predictors of LVH. Blood pressure control was also a significant factor, with poorly controlled hypertension increasing the odds of developing LVH by a factor of 2.3 (OR = 2.3, 95% CI 1.6-3.2). These results are presented in Table 6 (see PDF). Patients with both hypertensive retinopathy and LVH had a significantly higher incidence of hospitalization due to cardiovascular events, such as heart failure and arrhythmias. Among those with both HR and LVH, 22% experienced at least one hospitalization during the 6-month follow-up period, compared to 8% in those with no HR or LVH. Table 7 (see PDF) summarizes the hospitalization rates in relation to the presence of HR and LVH.

## Discussion:

In our study, we observed a statistically significant association between severity of hypertensive retinopathy and the presence of left ventricular hypertrophy, with higher grades of HR more frequently coinciding with LVH. This finding resonates with several previous investigations, yet also contrasts with some, highlighting the heterogeneity of evidence in this domain. Early work by Dahlöf *et al.* (1995) [12] demonstrated a positive correlation between retinal vascular changes and increased left ventricular wall thickness in patients with untreated hypertension, suggesting that retinal microvascular damage might reflect hypertensive cardiac remodeling. Similarly, an often cited study by Zoccatelli *et al.* (2025) [13] found a "strongly significant correlation between the degree of left ventricular mass index and the severity of hypertensive retinopathy and renal damage," reinforcing the notion that retinopathy and LVH are intertwined manifestations of systemic hypertension-induced target- organ damage. More recently, in a cross-sectional study by Varghese *et al.* (2016) [14], authors reported that grades 3 and 4 HR (advanced retinal changes) were significantly associated with LVH on echocardiography as well as ECG strain patterns, suggesting microvascular retinal changes may mirror myocardial structural changes. These observations support the hypothesis that hypertensive retinopathy could serve as a convenient, non invasive marker for cardiovascular target organ damage. However, the evidence is not unanimous. A study by Kabedi *et al.* (2014) [15], conducted among hypertensive patients in the Democratic Republic of the Congo, found no significant association between hypertensive retinopathy and LVH, chronic kidney disease, or cerebrovascular disease. In this context, our study adds to the body of evidence suggesting a meaningful association between retinopathy and LVH, particularly when HR is more advanced. This supports the potential utility of fundoscopic examination as part of routine evaluation in hypertensive patients to flag those at higher risk of cardiac target organ damage. Nonetheless, given the inconsistent findings across studies, these results should be interpreted cautiously and there remains a need for larger, preferably longitudinal studies across diverse populations with standardized definitions and rigorous adjustment for confounders.

## Conclusion:

We show a significant association between hypertensive retinopathy and left ventricular hypertrophy in hypertensive patients, suggesting that retinal changes may reflect underlying cardiovascular damage. The findings support the potential of hypertensive retinopathy as a non- invasive marker for monitoring cardiovascular risk. Further longitudinal studies are needed to confirm these results and better understand the mechanisms linking retinal and cardiac damage in hypertension.
